# Bilateral Trade Welfare Impacts of India’s Export Ban of Non-Basmati Rice Using the Global Partial Equilibrium Simulation Model (GSIM)

**DOI:** 10.3390/foods13193124

**Published:** 2024-09-30

**Authors:** Eihab Fathelrahman, Raeda Osman, Dana Loyd Keske Hoag, Gregory N. Sixt, Kenneth Strzepek

**Affiliations:** 1 Department of Integrative Agriculture, College of Agriculture and Veterinary Medicine, United Arab Emirates University (UAEU), Al-Ain P.O. Box 15551, United Arab Emirates; raeda_mohammed@uaeu.ac.ae; 2 Agricultural and Resource Economics Department, Colorado State University, Fort Collins, CO 80523, USA; dana.hoag@colostate.edu; 3 Abdul Latif Jameel Water and Food Systems Lab (J-WAFS), Massachusetts Institute of Technology (MIT), Cambridge, MA 02142, USAstrzepek@mit.edu (K.S.); 4 International Food Policy Research Institute, Washington, DC 20005, USA

**Keywords:** food trade, trade barriers, rice, partial equilibrium global simulation model (GSIM)

## Abstract

India, the world’s leading rice exporter, banned the export of non-Basmati white rice, accounting for 25% of its total exports (or 10% of the global rice trade). The ban aims to ensure availability to domestic Indian consumers and reduce domestic market prices, impacting global rice market accessibility, consumers, and producers across twelve regions. The study utilized the global simulation model (GSIM) to analyze the effects of trade restrictions on industries. The model uses national product differentiation to assess trade policy changes at global, regional, or national scales. It examined importer and exporter effects on trade values, tariff revenues, exporter surplus, and importer surplus. It found that India’s Voluntary Export Restraint (VER) ban on non-Basmati rice resulted in a higher local price and a negative global net welfare impact of USD 1.7 billion. The losses decreased to USD 1.4 billion when importing countries responded by reducing rice import tariffs by 25% and USD 1.1 billion when importing countries reduced tariffs by 75%. Sub-Saharan Africa, the Middle East, North Africa, and the Gulf Cooperation Council regions were most affected. The study also found minimal impact on consumer surplus in India due to inelastic rice demand.

## 1. Introduction

Rice is a food staple for more than 3.5 billion people worldwide, particularly in Asia, Latin America, and Africa. It is an important source of carbohydrates, vitamins E and B5, thiamine, calcium, folate, and iron. Rice is vital for food security, as about 95% of the global output of rice is produced and consumed in developing countries [1]. According to the United States Department of Agriculture (USDA) [2], the primary rice production for 2020/2021 was 148.30 million tones (Mt) in China, followed by 120.00 Mt in India, 35.30 Mt in Bangladesh, 34.90 Mt in Indonesia, and 27.10 Mt in Vietnam.

**Figure 1:**
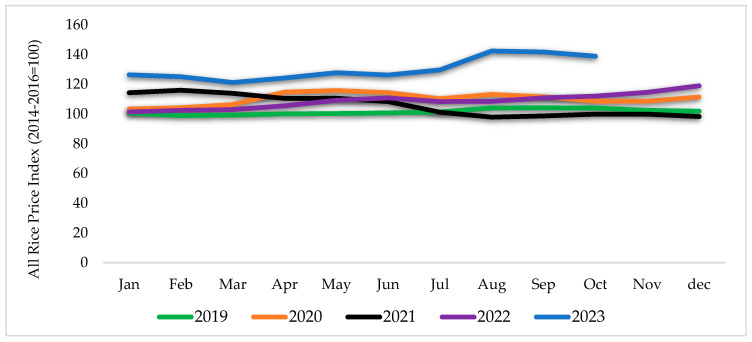
All Rice Price Index (Based on FAO Rice Price Index). Source: Food and Agriculture Organization (FAO) retrieved from https://www.fao.org/markets-and-trade/commodities/rice/fao-rice-price-update/en/, accessed on 20 October 2023) [4].

According to the Organization for Economic Co-operation and Development and the UN Food and Agriculture Organization (OECD/FAO), by 2032, the world is predicted to produce 577 million tons of rice, a 55-million-ton increase, primarily in Asian nations, with India leading the way. China, the world’s largest rice producer, is expected to increase production at a similar rate as in the past decade, mainly due to yield improvements [3]. Between 2018 and 2022, the reference export price for milled rice (FAO All Rice Price Index adjusted to India 5%) fluctuated within a small range between USD 387/t and USD 420/t. Figure 1 shows that international rice prices reached 142 points in August 2023, their highest level since October 2011, partly because of limited exportable supply brought on by production declines in some significant exporting countries. Demand from the Far East, Africa, and the Middle East is expected to expand over the medium term. Still, exporter supply growth is anticipated to slightly boost nominal prices to USD 459/t by 2032 [3].

This sharp increase in international rice prices could be due to geopolitical conditions, El Niño weather impacts, and extreme climatic conditions in the rice-producing countries [5]. As rice is one of the commodities sensitive to El Niño, past episodes were associated with 4–11% decreases in rice yields [6]. Trade restrictions and related policies, such as export bans and licensing requirements, contributed to the increase in the global rice price [5].

**Figure 2:**
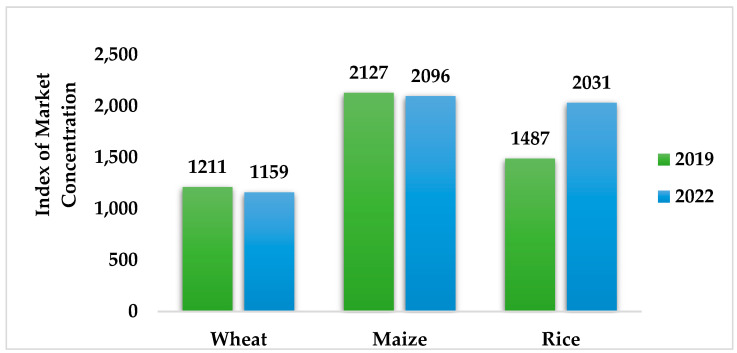
Index of market concentration in grain production. Source: Organization of Economic Co-operation and Development; OECD-FAO Agricultural Outlook 2022–2031, OECD Agriculture statistics (database), retrieved from: http://dx.doi.org/10.1787/agr-outl-data-en; U.S. Department of Agriculture; World Bank (accessed on 21 October 2023) [3].

According to the World Bank’s projection for commodities [7], market concentration could be a factor in fluctuating prices. A typical instance is the rice market. According to the Herfindahl–Hirschman Index (HHI Index), a widely used indicator of market concentration, the HHI Index can range from close to 0 to 10,000, with lower values indicating a less-concentrated market. Rice markets changed over the past three years from competitive to somewhat consolidated (Figure 2).

**Figure 3:**
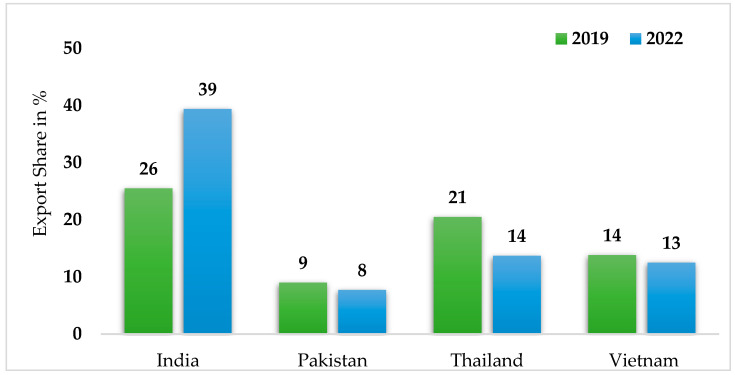
Export shares of major rice-producing countries in percentage. Source: Organization of Economic Co-operation and Development; OECD-FAO Agricultural Outlook 2022–2031, OECD Agriculture statistics (database), http://dx.doi.org/10.1787/agr-outl-data-en; U.S. Department of Agriculture; World Bank (accessed on 21 October 2023) [3].

India is now the top rice exporter, replacing Thailand. When compared to Thailand, India’s exports increased while Thailand’s exports decreased, increasing India’s market share of rice exports from 25% in 2019 to 40% in 2022 (Figure 3). Such a change in market concentration might put upward pressure on prices due to the periodic trade restrictions imposed by India, the most recent of which is a 20% export duty imposed on some rice varieties and a complete ban on broken rice [3].

**Figure 4:**
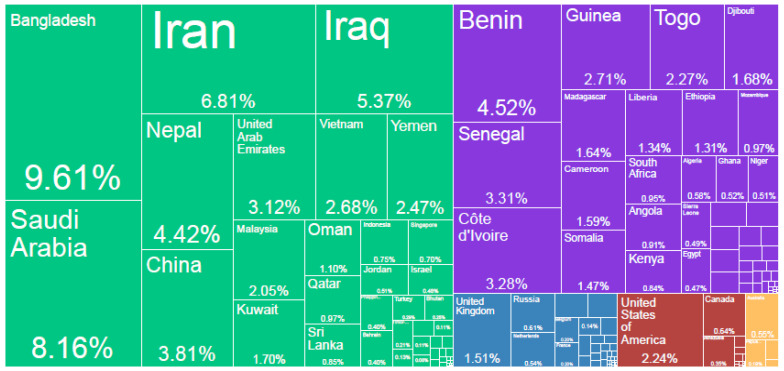
Geographical map displaying India’s rice import partners in 2021. Source: The Atlas of Economic Complexity by Harvard growth lab, retrieved from https://atlas.cid.harvard.edu/explore?country=104 (accessed on 5 November 2023) [8].

Figure 4 shows the countries importing Indian rice. The markets highly exposed to India’s export restrictions are major importers of Indian rice concentrated in Asia, Sub-Saharan Africa, and the Middle East and North Africa (MENA) region.

On 20 July 2023, India banned the exports of non-Basmati rice, which constitutes about 25% of total rice exported from the country, with immediate effect to ensure adequate availability of non-Basmati rice in the Indian market and to cap the rise in prices in the domestic market [9]. This might reduce the global rice supply and exacerbate the price increase. In the long run, affected importers may turn to alternative suppliers.

For similar reasons, on 9 September 2022, India imposed a 20% export levy on some types of rice to safeguard its local food supply. During the 2007 food price crisis, India adopted similar moves to prohibit rice exports, pushing global rice prices to new highs [10]. The study examined how India’s rice export tariffs influence rice trade, production, pricing, producer surplus, consumer surplus, and subsidy adjustments in other countries. It revealed that India’s trade volume in rice exports fell by over 80%, decreasing China’s rice purchases from third-party markets. Tariffs have little impact on consumer prices in India and China, as importers bear the cost. The tariffs did not provide significant protection for Indian rice buyers, resulting in a 24.4% decrease in output. Tariffs also benefit India’s social net welfare while harming China, but they significantly negatively impact Indian rice farmers’ and consumers’ welfare.

A study by [11] examined the implications of rice trade policy change in the Philippines using a global rice partial equilibrium model. They addressed three major concerns: agricultural price heterogeneity among 16 regions due to tariffs and productivity increases, import differential by origin due to various tariffs, and domestic price implications in other countries. The analysis revealed that the policy will boost imports by around 21% in 2019. The authors proposed that the change will benefit low-income customers and that policymakers should utilize the increased tariff money to assist rice producers in improving their competitiveness or shifting to other crops.

As a major rice grower and exporter, India has a significant presence in the worldwide rice market. In 2008, the Indian government introduced protectionist trade measures, resulting in an estimated USD 260 million boost in national income. However, this approach encountered issues since it diminished global welfare at the price of increasing national welfare. Trading partners objected to these policies and sought methods to offset the fall in welfare or responded with their protectionist measures. After India prohibited all non-Basmati rice exports, other major exporters, such as Thailand and Indonesia, followed, resulting in price instability and shortages in other nations. As with those from big importing countries, India’s customers are unaffected by global pricing. Export restrictions may significantly affect India and rice growers in the long run. They may increase smuggling and hoarding, reduce trade prospects, and render India’s production-oriented aid packages economically unsustainable to continue. High public-sector debt also reduces capital investment. Meeting future rice demand will be difficult owing to increased competition for land, water, and environmental damage. While protectionist trade policies are profitable in the short run, their long-term consequences are unsustainable [12].

The primary purpose of this study is to simulate the impact of India’s non-Basmati rice export ban on the producer surplus, consumer surplus, and net welfare of India and major rice-importing countries and regions of the world. The study then simulates the importing countries’ efforts to overcome India’s non-Basmati rice export ban, identifying alternative rice-exporting countries such as East Asian Pacific countries.

## 2. Literature Review

According to data collected by the World Bank and Global Trade Alert, 135 policy measures affecting food and fertilizer trade were announced or implemented between the beginning of 2022 and 2 June 2022. Some were restricting, while others were liberalizing, such as eliminating a previously enforced measure. The vast majority (74 measures) restrict exports, with two-thirds being outright bans on exports. As of 2 June 2022, 86 countries were responsible for increased trade-related regulations imposed on agricultural goods and fertilizers since the beginning of the year, mainly in Europe and Central Asia. A total of 34 nations implemented restricted export controls for food and fertilizers. This figure is nearing that of the 2008–2012 food crisis, when 36 nations implemented export restrictions, resulting in price increases of major commodities such as wheat and rice by more than 30% [13]. According to [14], large food-exporting nations’ implementation of food protectionist policies harms food-importing countries while increasing global food prices. Studies examining the immediate impact of the 2007–2008 food price increase generally used the net benefit technique to evaluate welfare effects [15]. Ivanic and Martin [16] estimated the welfare impacts of the 2007–2008 global food price increase for nine low-income countries, assuming perfect transmission of global and local prices. They discover that high global food costs exacerbate poverty, particularly in metropolitan regions.

Sonia Akter reviewed the effect of food export restrictions on local food prices and the welfare of food system actors in exporting nations. While these restrictions might temporarily reduce price increases by increasing short-term domestic supply, they frequently have unforeseen negative implications for food producers. The net welfare effect was determined by the percentage of net food purchasers in the economy, price changes between producers and consumers, and the importance of limited food products to family income and spending. Without a large producer price support scheme, the short-term welfare benefit is negligible and especially bad for rural residents. Export limitations also prohibit domestic farmers from accessing high international agricultural commodity prices, resulting in substantial economic costs, such as lost producer revenue, agricultural investments, enforcement expenses, and fiscal costs [17]. Grain export restrictions during COVID-19 risk food insecurity in many low- and middle-income countries. The effects of COVID-19 on international agricultural supply chains and locusts destroying crops and livelihoods in the Horn of Africa and South Asia threatened global food security. A study by [18] estimated the potential impact on global wheat, rice, and maize supply and pricing. The Authors concluded that local production decreases have a minor influence on worldwide pricing and supply. However, trade restrictions and cautious purchases by a few important actors might cause global food price spikes and catastrophic local food shortages.

The study by Thomas et al. [19] examined the impact of grain harvest failures and temporary export restrictions on national and international food security. The study used the AGLINK-COSIMO model to analyze the situation. Results show that limited grain exports from Ukraine and Kazakhstan significantly increased prices due to the situation. Temporary restrictions can exacerbate the situation on world grain markets, particularly for grain net importing countries. Introducing export restrictions could decrease domestic consumer prices for countries such as Ukraine, where exports are a significant part of their total grain production. The study highlights the need for greater cooperation between exporting countries to prevent importing countries from being denied necessary grain supplies. Similarly, Fan Feng et al. [20] studied the impact of the Russia–Ukraine conflict on global food security using sector linkages and the quantitative general equilibrium trade framework. They found that the conflict would lead to soaring agricultural prices, decreased trade volume, and severe food insecurity, particularly for countries heavily reliant on grain imports from Ukraine and Russia. Major production countries such as the United States and Canada may benefit from the conflict, while restrictions on upstream energy and fertilizer could exacerbate the negative effects of food insecurity.

Chen and Zhao [21] published “Understanding Global Rice Trade Flows: Network Evolution and Implications.” The authors analyzed the structural evolution of the global rice trade from a network perspective to better understand the international rice trade supply chain and its implications on global food security from 2000 to 2021. The author showed that the global rice trade networks increased density, characterized by Asia as the primary export source and Africa as an important import market. From a regional perspective, the authors indicated that regional backbone structures revolve around India as the core, Thailand and Pakistan as the second cores, and critical nodes to the regional rice trade are represented by Italy, the United States, China, and Vietnam.

In parallel, Digvijay S. Negi [22] examined the welfare effects of rising rice and wheat prices on Indian households. The study highlighted the enormous variation in such consequences and observed that high rice and wheat prices were generally helpful to farming households. A significant and overwhelming positive income effect for net producer families also drove this total benefit. Both net producers and consumers could withstand an increase in their overall cereal spending and food budget but in different ways. Net consumers mainly substituted market-purchased rice and wheat with home-produced rice, wheat, and other cereals. Meanwhile, net producers substituted market-purchased rice and wheat with PDS rice and wheat, among other cereals. Subsidized PDS rice and wheat were important in stabilizing total food consumption in net consumer and producer families, although net producer households benefited the most. Finally, we detected some indirect consequences in net producer households, such as greater total workdays and increased usage of family labor on their farm.

Wailes [23] reviewed global rice market models, emphasizing the importance of quantitative models and their ability to predict market dynamics. These models are typically part of a multicommodity framework, reflecting demand and supply dynamics. However, capturing government intervention’s heterogeneous and creative nature in the market is crucial. Disaggregated commodity space and key exporting and importing countries/regions are desirable. Understanding model results is crucial for evaluating decisions without unrealistic reliance on any single model. Internal and external model validation is essential for quantitative models. While models are not always correct in projections, achieving success in market and policy effects is the standard for all models, providing practical outcomes and guidance for policymaking.

Using a partial equilibrium model, [24] calculated the short- and long-term impacts of COVID-19 and rice policy on Bangladesh’s rice market and food security. The results demonstrate mixed implications for food security in FY 2019/20, with a 5% increase in food availability and a 17% drop in food stability. Policy simulations reveal that higher import tariffs increase self-sufficiency but reduce rice supply and accessibility. Closing the current yield gap increases rice availability and accessibility, but it may be riskier and more expensive. The findings help to understand strategies targeted at attaining sustainable development goals and increasing resilience to future shocks such as COVID-19. Similarly, Kozicka et al. [25] used a two-commodity dynamic partial equilibrium model with stochastic shocks to investigate existing and prospective reforms in Indian food policy for staple cereals, wheat, and rice. The model is empirically based and accurately reproduces historical values. The research assessed the execution of the National Food Security Act (NFSA) using various policy tools and two regime transition scenarios: cash transfers and deficit payments. The effects on market fundamentals and fiscal costs were simulated in the medium term till 2020/21. The study emphasized that the NFSA pressured fiscal expenses and public stocks significantly. Policy measures affecting procurement prices may result in more significant fiscal costs with lower but more erratic pricing. A cash-based system could provide significant savings and reduce fiscal expenditures, mainly if targeted at people experiencing poverty while leaving sufficient stocks due to higher private stocks.

The World Integrated Trade Solution (WITS) partial equilibrium model was used by Fathelrahman et al. [26] to assess the welfare effects of food trade liberalization in India, Egypt, Pakistan, Saudi Arabia, and the United Arab Emirates. Macroeconomic parameters, domestic policy objectives, and food security indicator data are utilized to evaluate the simulations’ effects on food supply and stability. The simulation findings for India, Egypt, and Pakistan show yearly welfare benefits (consumer surplus) of USD 2571, 340, and 25 million, respectively. In contrast, Saudi Arabia and the UAE have gains of USD 14 and 17 million. The results suggest that eliminating tariffs would have a wide-ranging welfare impact on food items in these nations. Furthermore, decreases in essential commodities directly linked to dietary energy and protein availability would directly impact low-income people. Lowering the highest tariffs on those items might increase the actual earnings of almost 350 million people by 7.5% or more, causing a shift in consumption toward more diverse and nutritionally sound diets. Comparably, Bayale et al. [27] estimated the possible trade, welfare, and tax effects of Ghana’s adoption of the African Continental Free Trade Agreement (AFCFTA) by utilizing the WITS-SMART simulation model using 2018 disaggregated international trade data. The study predicted that overall trade impacts in Ghana would rise by USD 148.3 million while increasing consumer welfare by USD 8.597 million. However, revenue losses were anticipated, as the country’s tariff revenue was expected to fall by USD 8.604 million. Overall, the free trade area was predicted to enhance the country’s trade balance, with exports outweighing imports. The study advised that the government should safeguard many tariff lines for sensitive and banned items to reduce revenue losses. Using the global simulation model (GSIM), this study intends to provide insight into the impact of food trade restrictions on a country’s welfare.

## 3. Trade Impact Model and Data Sources

The following describes the Global Simulation (GSIM) Model, data sources, and scenario assumptions.

### 3.1. The Global Simulation Model (GSIM) Model

To address the impact of India’s rice export ban on global welfare, the global simulation model (GSIM) is applied to analyze global, regional, or unilateral trade policy changes [28,29]. The model is an imperfect substitute of world trade employing a partial equilibrium approach. The results of the GSIM allow the assessment of importer and exporter effects related to tariff revenues, producer surplus, consumer surplus, and changes in overall domestic prices. The model requires the input of a bilateral trade matrix at world prices, an initial matrix of bilateral import tariffs in ad valorem form, a final matrix of bilateral import tariffs in ad valorem form, export supply elasticities, aggregate import demand elasticities, and elasticities of substitution.

This model was used in several trade analysis publications, especially trade bans. For instance, GSIM [30] examined the trade implications of gene-edited wheat in five regulatory situations. The first scenario is that Canada does not commercialize gene editing, but Australia and the United States do. The second scenario depicts Canada commercializing gene editing, while the United States and Australia do not. The third scenario has the three exporters commercializing gene-edited wheat. Countries with strict rules, such as Italy and Algeria, prohibit the importation of gene-edited wheat. The fourth scenario is that only Italy imposes a trade prohibition on Canadian gene-edited wheat, and only Canada commercializes it. The fifth scenario is that Canada, the United States, and Australia are selling gene-edited wheat, while formerly strict nations such as Italy and Algeria are becoming willing to import it. According to [31], the GSIM can simultaneously analyze the impacts of policy changes on trade in domestic and foreign markets since it enables disaggregated sector-specific analysis within a global framework while also assisting in predicting self-sufficiency, a major policy concern in food markets.. The study extended and used the GSIM to analyze policy changes’ impact on domestic and international fish and seafood trade. Likewise, ref. [32] employed the “Global Simulation Analysis of Industry-Level Trade Policy” (GSIM) model to assess the impact of the ‘without-US’ Trans-Pacific Partnership (TPP) agreement on Vietnam’s clothing sector. It looked at two scenarios: the TPP11 without the United States and the likely dead TPP with Vietnam joining the Regional Comprehensive Economic Partnership’s alternative free trade agreement (FTA). The findings demonstrate that if the United States withdrew from the TPP, Vietnam’s clothing industry’s export value and trade welfare would fall considerably compared to the entire TPP12. However, if Vietnam entered the RCEP, compliance with origin regulations is possible, which might somewhat boost Vietnam’s garment exports into the FTA. Similarly, to investigate the relevance of production capacity restrictions in international trade policy analysis, [33] used partial equilibrium GSIM and simulated the impact of a restriction on orange juice imported from Brazil in 2011. The findings report that a partial ban might raise US domestic orange juice prices by 0.2% and boost producer surplus by USD 1.2 million without a production capacity constraint. However, assuming an almost entirely inelastic supply, the partial prohibition might raise the price by 6.1% and reduce the producer surplus by USD 36 million. This emphasizes the need to take production capacity restrictions into account while analyzing empirical trade policies.

#### 3.1.1. Demand (Imports) and Supply (Exports) Elasticities

Francois and Hall [28,29] estimate calibrated values, cross-price elasticities, and own-price elasticities through the import demand equation. The import demand of a product *I* within an importer country *v* from an exporter country *r* is a function of the industry prices and the total as presented in Equation (1), where $Y_{i,v}$ is the total expenditure in country *v* on imports of *i*, $P_{i,v,r}$ is the export price for goods from an exporter *r* to an importer country *v*, the price of other varieties representing the price of rice from other sources *r*. In demand theory, this results in weak separability.

(1) $$M_{i,v,r}=\int (P_{i,v,r},P_{\left(i,v\right),s\neq r},Y_{i,v})$$

Francois and Hall [28,29] applied the Slutsky decomposition of partial demand and took the zero homogeneity of Hicksain demand to derive the own- and cross-price elasticity. The cross-price elasticity is presented in Equation (2).

(2) $$N_{\left(i,v\right).\left(r,s\right)}=\theta_{\left(i,v\right),s}\;(E_{m}+E_{s})$$

where $\theta_{\left(i,v\right),s}$ is the expenditure share of product *i* imported by importer country *v* from an importing country *s*. $E_{m}$ is the elasticity of composite demand in importing country *v* and $E_{s}$ is the elasticity of substitution. The own-price elasticity is presented in Equation (3)

(3) $$N_{\left(i,v\right)\left(r.r\right)}=\theta_{\left(i,v\right),r}E_{m}-\sum_{s\neq r}\theta_{\left(i,v\right),s}E_{s}=\theta_{\left(i,v\right),r}E_{m}-\left(1-\theta_{\left(i,v\right),r}\right)E_{s}.$$

Next, specify the fundamental Supply and demand equation. $P_{i,r}^{*}$ is defined as the export price received by exporter *r* and $P_{\left(i,v\right),r}$ is the internal price for the same good *r* (within the exporting country). Then, the price for the same goods can be linked as follows in Equation (4)

(4) $$P_{i,v,r}=\left(1+t_{\left(i,v\right),r}\right)P_{i,r}^{*}=T_{\left(i,v\right),r}P_{i,r}^{*}.$$

White $T=\left(1+t\right)$ is the power of the tariff (the proportional price markup achieved by the tariff t).

Next, Francois and Hall [28,29] define the export supply equation in an exporter country *r* as a function of the world price as in Equation (5)

(5) $$X_{i,r}=\int (P_{i,r}^{*}).$$

The reduced form of the individual (national) demand and supply equations, Equations (1), (4) and (5) are differentiated and rearranged to Equations (6)–(8) (Francois and Hall [28,29])

(6) $${\hat{P}}_{\left(i,v\right)r}={\hat{P}}_{i,r}^{*}+{\hat{T}}_{\left(i,r\right),r}$$

(7) $${\hat{X}}_{i,r}=E_{\left(i,r\right)}{\hat{P}}_{i,r}^{*}$$

(8) $${\hat{M}}_{\left(i,v\right),r}=N_{\left(i,v\right)\left(r.r\right)}{\hat{P}}_{\left(i,v\right),r}^{*}+\sum_{s\neq r}N_{\left(i,v\right),\left(r,s\right)}{\hat{P}}_{\left(i,v\right)s}$$

where ^ denotes a proportional change so that $\hat{x}$ = $\frac{dx}{x}$.

#### 3.1.2. Global Equilibrium Conditions

Francois and Hall [28,29] aim to arrive at a model defined in terms of world prices P*.

Substituting Equations (2), (3), and (6) into (8) and summing over import markets results into Equation (9).

(9) $${\hat{M}}_{i,r}=\sum_{v}N_{\left(i,v\right),\left(r,r\right)}{\hat{P}}_{\left(i,v\right)r}+\sum_{v}\sum_{s\neq r}N_{\left(i,v\right),\left(r,s\right)}{\hat{P}}_{\left(i,v\right)s}=\;\sum_{v}N_{\left(i,v\right),\left(r,r\right)}\left[P_{r}^{*}+{\hat{T}}_{\left(i,v\right),r}\right]+\sum_{v}\sum_{sr}N_{\left(i,v\right),\left(r,s\right)}+\left[P_{s}^{*}+{\hat{T}}_{\left(i,v\right),s}\right]$$

Equation (9) is equal to the modified version of Equation (7), yielding each export variety’s global market clearing condition [28,29].

$${\hat{M}}_{i,r}={\hat{X}}_{i,r}$$

(10) $$E_{x\left(i,r\right)}{\hat{P}}_{i,r}^{*}=\sum_{v}N_{\left(i,v\right),\left(r,r\right)}{\hat{P}}_{\left(i,v\right)r}+\sum_{v}\sum_{sr}N_{\left(i,v\right),\left(r,s\right)}{\hat{P}}_{\left(i,v\right)s}=\;\sum_{v}N_{\left(i,v\right),\left(r,r\right)}\left[P_{r}^{*}+{\hat{T}}_{\left(i,v\right),r}\right]+\sum_{v}\sum_{sr}N_{\left(i,v\right),\left(r,s\right)}+\left[P_{s}^{*}+{\hat{T}}_{\left(i,v\right),s}\right]$$

Equation (10) is the core equation for the system implemented in the Excel spreadsheet. Spreadsheet Equation (10) is used to back solve for import quantities [28,29]. The welfare effects are estimated: the producer surplus **Δ*PS*** (the exporter surplus) and the consumer surplus (the importer surplus) **Δ*CS***.

#### 3.1.3. Welfare and Revenue Effects

**Figure 5:**
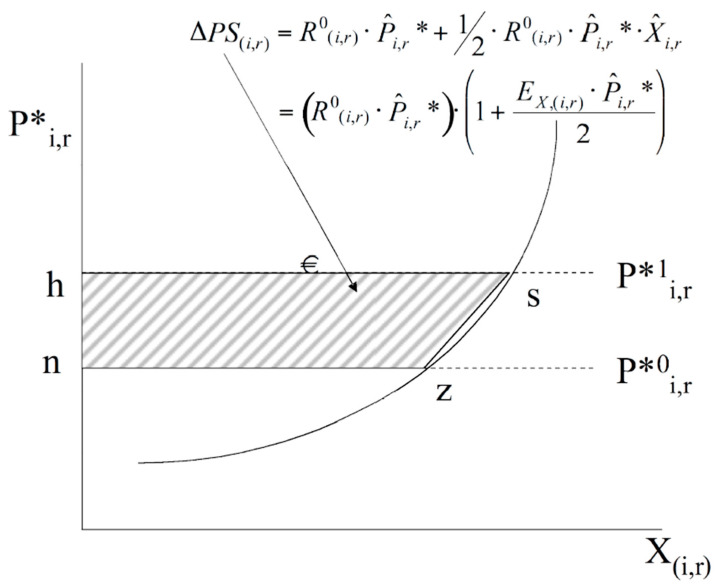
Export Markets and Producer/Exporter Surplus Measures. Source: Joseph Francois and H. Keith Hall, 2003. “Global Simulation Analysis of Industry-Level Trade Policy: the GSIM model”, IIDE Discussion Papers 20090803, Institute for International and Development Economics [28,29].

The measurements of producer surplus are adopted from Francois and Hall [28,29] and presented conceptually as the area of the trapezoid *hszn* in Figure 5, which is translated into Equation (11).

(11) $${\Delta PS}_{\left(i,r\right)}=(R_{\left(i,r\right)}^{0}\times {\hat{P}}_{i,r}^{*}).\;(1+\frac{E_{x\left(i,r\right)}{\hat{P}}_{i,r}^{*}}{2})$$

where *R* is the benchmark export revenues valued at world prices and ${\hat{P}}_{i,r}^{*}$ is the percentage change in the world price of goods *i* exported from country *r*.

**Figure 6:**
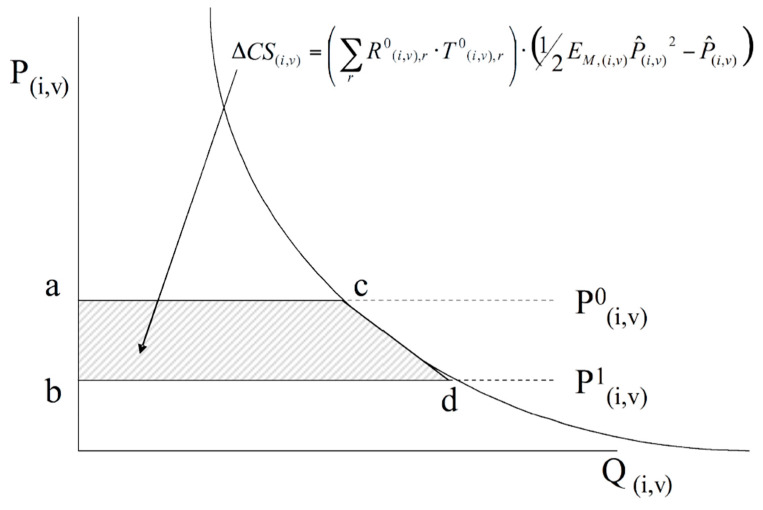
Imports markets and consumer surplus measures. Source: Joseph Francois and H. Keith Hall 2003. “Global Simulation Analysis of Industry-Level Trade Policy: the GSIM model”, IIDE Discussion Papers 20090803, Institute for International and Development Economics [28,29].

Meanwhile, the change in consumer/importer surplus is calculated as the area, and in Figure 6, this can be expressed as the Equation (12) as follows:

(12) ΔCSi,v=∑rRi,v,r0·Ti,v,r0×12EM,i,vP^(i,v)2−P^(i,v)

### 3.2. Data Source and Assumptions

The data required to run the GSIM include trade values by origin and destination, export tax, composite demand, supply, and substitution elasticity. The year 2021 was chosen as the base year for the analysis as it is the most recent and representative of the global equilibrium market with complete data for the case studies. All the data reported correspond to the HS four-digit level (1006), which refers to rice. The countries analyzed in this study are in Table 1:

The trade flow in 2021 serves as a benchmark for the GSIM model. Table 2 shows the trade flows between the regions of interest before India’s export prohibition. The values utilized are those reported by the importers. Traded values of rice between countries in US dollars in thousands are extracted from the United Nations Statistics Division Commodity Trade (COMTRADE) through access to the WITS database. This study used the ad valorem form, tariffs applied by importing countries available in the United Nations Conference on Trade and Development (UNCTAD) Trade Analysis Information System (TRAINS) through access to the WITS database. Table 3 shows the initial tariff used in the model, which is entered in the form of T = 1 + t, where t is the rate of the tariff markup relative to the world price. The final export tax representing the trade ban is entered as S = 1/(1 + t), where t is the tax rate imposed by the exporter; 25% in this case.

The model also requires three types of elasticities: the aggregate import demand elasticities are borrowed from Kee et al. and revisited by Grübler et al. [36], as shown in Table 4. The elasticity of substitution of value 5 is adopted for all countries [28,29]. An elasticity of export supply equal to 1.5 was adopted (Table 4) by Francois and Hall [28,29] and used for major rice exporters (India, the East Asia-Pacific region, the South Asia region, and the US). The assumption of “a small country” was adopted from Holzner [37] regarding the rest of the countries. Holzner assumes that when a country is not considered a major exporter of a good, the value 0.5 is used for its export supply elasticity. Similar values of export supply elasticities, import demand elasticities, and substitution elasticities were used by [30,32,33].

The required inputs to run the GSIM model are the matrix of bilateral trade at world prices, the initial matrix of the import tariffs applied bilaterally in the ad valorem form, the final matrix of the bilateral tariffs in the ad valorem form for rice, the initial and final export tax, and the sets of the elasticities (Table 4).

### 3.3. Modeling the Trade Effects of India’s Rice Export Ban

The GSIM is particularly suited for simulating multiple countries’ tariff or export tax changes. However, the GSIM in its original form does not allow users to conduct simulations related to trade bans. The model was modified by [33] to make it suitable for the trade ban and production capacity limit. The modification was accomplished by applying an increase in the tariff rate on the affected country, large enough to reduce the country’s exports to the United States to zero (such price is known as virtual or choke price). The result was achieved by adding the corresponding tariff rate (final tariff rate, not the baseline one) as a new variable and a new constraint of zero imports from the affected country. According to [33], a partial ban or a ban for multiple countries can also be conducted in a similar fashion. To model a country’s production capacity limit, an export tax was placed on the country of interest so that the new production following a policy change, either a tariff change or a trade ban, does not exceed a certain level. This was achieved by adding the corresponding export tax rate (final rate, not the baseline one) as a variable and a new constraint of production capacity limit.

In this study for modeling purposes within the GSIM, the trade ban is modeled as an export tax increase to mirror the new trade policy, effectively reducing the rice export from India. According to the Press Information Bureau of the government of India, the banned non-Basmati white rice accounts for 25% of the total rice exports by India [9]; relying on this and for simplicity purposes, this study modeled the ban as an increase in export tax by 25%, which reduced the imported quantity of rice from India by almost 25%.

### 3.4. Global Simulations of India Rice Export Ban

The following shows how scenarios are set in this study:

Scenario 1 (Base Scenario)—India Export Ban of Rice (Voluntary Export Restriction): India imposes an export ban on non-Basmati rice. The trade ban is modeled as an imposition of India’s 25% export tax on its trading partners, representing banning non-Basmati only [9]. Countries continue to trade with an initial export supply elasticity of 1.5 for India, East Asia-Pacific, South Asia, and the USA, and 0.5 for the rest of the countries and regions included in this study. This scenario closely mimics what happened due to India’s rice export ban and will serve as a baseline for the following two scenarios.

Scenario 2: Importing Countries Respond by Reducing Rice Imports Tariff from Non-Indian Countries by 25%: This scenario assumes countries and regions will respond to the export ban by reducing import tariffs. In this scenario, we applied a 25% tax on Indian non-Basmati rice exports tax as a proxy for the trade ban and a 25% reduction in the final import tariff as a response by the importing countries. The tariff reduction does not include India, as it already imposed an export ban.

Scenario 3: Importing Countries Respond by Reducing Rice Imports Tariff from Non-Indian Countries by 50%: In scenario 3, we assume that countries and regions will be more open and reduce their final import tariff for rice by 50% for non-Indian exporters. We assume India continues the policy described in Scenario 2 (25% export tax).

Scenario 4: Importing Countries Respond by Reducing Rice Import Tariffs from Non-Indian Countries by 75%: In scenario 4, we expect nations and regions to become more open and cut their ultimate import tariffs on rice by 75%, except for India. India would maintain the export restriction at the same level (25% export tax).

## 4. Results

The outcomes of the four scenarios that were simulated in the GSIM are shown in this section. Tables showing the change in net welfare, expressed in thousands of dollars, and the percentage change in prices and output are used to present the outcomes of each scenario.

### 4.1. Scenario 1: India Rice Export Ban as Voluntary Export Restraint (VER)

Table 5 shows the estimates of the impact of India’s export ban on non-Basmati rice on world welfare. The impact was simulated by applying a 25% export tax in the model. Our result suggests that India experiences over USD 1 billion in losses in producer surplus and net welfare under this scenario. The reduction in producer prices by 17% in India explains the producer surplus loss. In comparison, there is a negligible negative change in consumer surplus of USD 77 thousand due to a small rise in overall consumer prices in India by 1%. Even with a domestic reduction in producer prices of 17%, India imports a significant amount of rice from the global market, where the world market price increased. Hence, there is an overall negative net welfare effect in India.

This scenario also shows that consumers in the rest of the world would experience losses of approximately USD 628 million. Importing countries, particularly in Sub-Saharan Africa (SSA), the Europe–Central Asia region, and Gulf Cooperation Council (GCC) Countries, are worse off, with welfare losses estimated at USD 353 million, USD 87 million, and USD 62 million, respectively. This is explained by the negative tariff revenue and increased consumer prices by 3% and 1%, leading to negative consumer surplus and net welfare (see Table 5). The ban lowered the quantity traded from India by more than 11% in most regions, showing India’s negative producer surplus. Table 6 shows that trade flows are shifting towards East Asia-Pacific, Europe–Central Asia, and the United States.

### 4.2. Scenario 2: Importing Countries Response to India Rice Export Ban (25% Reduction in Tariffs)

This scenario assumes countries and regions will respond to the export ban by reducing their rice import tariffs by 25%. Our results project that India experiences a positive consumer surplus estimated at USD 423 thousand due to a drop in world market prices, leading to a 6% drop in Indian consumer prices. Furthermore, negative net welfare is more significant than USD 1 billion due to the over USD 1 billion loss in producer surplus. Consumers in Canada, European Union (EU) countries, the United Kingdom, and the GCC countries do not gain from the retaliation since their initial rice import tariffs were zero, decreasing consumer surplus and negative net welfare (Table 7). China, Europe–Central Asia, Middle East–North Africa (Exc. GCC), and Sub-Saharan Africa experience rising consumer surplus.

However, they experience negative net welfare due to a loss in tariff revenue caused by the tariff reduction assumption. East Asia-Pacific and South Asia experienced a positive change in net welfare due to the new trade opportunities created, as evidenced by an increase in the quantity traded displayed in Table 8. This results from an increased producer surplus greater than the negative consumer surplus and tariff revenue losses. The initial import tariff of the United States was low. Hence, a 25% tariff reduction had a minor impact, judging by a negative consumer surplus, tariff revenue, and net welfare. Conversely, the tariff reduction in the Latin America–Caribbean region, which has high rice import tariffs, resulted in a positive consumer surplus and net welfare despite the reduced tariff revenue.

### 4.3. Scenario 3: Importing Countries Response to India Rice Export Ban (50% Reduction in Tariffs)

Similar to scenario 2, we suppose that countries and regions will be more open and reduce their final import tariff for rice by 50%, except for India. The impact is significantly increased compared to a 25% tariff reduction (Table 9). India’s producers are still experiencing inelastic supply due to the export ban, resulting in a loss of USD 1.1 billion of producer surplus, while consumer surplus increases a negligible USD 996 thousand. The result is a significant loss in net welfare.

In another aspect, rice-producing and exporting countries and regions, particularly East Asia-Pacific and South Asia, show increases in welfare due to the reductions in tariffs by the rest of the world, resulting in increased exports. This welfare increase is due to increased demand and market opportunities as most global regions redirect trade flow to East and South Asia, as shown in Table 10. Despite the loss of tariff revenue in Latin American and Caribbean countries, the 50% duty reduction increased from a consumer surplus of USD 369 thousand and a net welfare of USD 119 thousand. For the world, net welfare decreases due to the significant impact of the loss of tariff revenue.

### 4.4. Scenario 4: Importing Countries Respond by Reducing Rice Import Tariffs from Non-Indian Countries by 75%

This scenario illustrates the impact of India’s non-Basmati rice export ban when importing nations respond by reducing rice import tariffs from non-Indian countries by 75%. As indicated in Table 11, the reduction in import tariffs resulted in negative government revenue in all nations and regions while creating a positive producer surplus except for India. India’s inelastic export supply reduced production and producer prices by 10% and 20%, respectively, resulting in a USD 1.2 billion loss in producer surplus and net welfare. On the contrary, reducing import tariffs would result in a 23% reduction in overall consumer costs, with Indian consumers experiencing limited benefits.

In this scenario, all countries had negative net welfare except rice-producing and exporting countries (e.g., East Asia-Pacific, Latin America–Caribbean, and South Asia). The positive net welfare in those countries is caused by a considerable increase in the producer surplus due to new trade opportunities, as shown by the change in the quantity traded in Table 12. On the other hand, the negative net welfare experienced by Canada, the European Union, and the GCC countries results from a negative consumer surplus.

## 5. Discussion

Food export restrictions, such as bans, taxes, quotas, or licensing, can benefit domestic food purchasers by stabilizing food prices while reducing food sellers’ net revenue. This is because staple food demand is price inelastic, and producers cannot reduce commodity supply. Government interventions in the market create uncertainty about prices and returns from agricultural investments, leading farmers to adjust their production decisions in the long run. This could decrease agricultural production and productivity, lowering long-term agricultural growth and income. The new policy’s estimated trade impacts are expressed in terms of changes in net welfare, tariff revenues, consumer surplus, producer surplus, and price changes. New trade values and welfare impacts are produced by the GSIM model, which also creates new prices that clear the markets. Changes in traded amounts are another aspect of the results that provide insight into the trade diversion that happens following changes in trade policies by certain nations. The GSIM simulated four scenarios to examine how India’s export prohibition affected net welfare, consumer and producer surplus, and government revenue in the selected 12 areas.

Scenario 1 shows the estimates of the impact of India’s export ban on non-Basmati rice on world welfare. India’s export ban on non-Basmati rice reduces producer surplus and net welfare. The ban lowered the amount traded from India by more than 11% in most regions, demonstrating a negative producer surplus. Although India’s rice exports decreased, domestic market prices rose, causing a certain degree of loss to consumer welfare. This indicates that the trade policies have a large negative impact on Indian rice producers’ and consumers’ welfare. These results are consistent with the findings aligned with Wenjie Li and Hongjin Xiang [10] and with the study’s findings on the effects of India’s trade policy on rice production and exports [12]. Additionally, India’s export ban on non-Basmati rice significantly negatively impacts consumer surplus and net welfare in the other selected regions. Adversly, Sub-Saharan Africa, East Asia–Pacific South Asia, and Europe–Central Asia.

The simulation findings from the second, third, and fourth scenarios, which assume that countries and regions would respond to the export ban by lowering their rice import tariffs to varying degrees, have the following predictions: First, India depicts a positive consumer surplus due to a decline in global market prices, but producers face inelastic supply due to the export ban, resulting in a considerable loss in net welfare. Second, some regions, such as China, Europe–Central Asia, Middle East–North Africa (excluding the Gulf Cooperation Council), and Sub-Saharan Africa, experience increasing consumer surpluses while having negative net welfare due to a loss in tariff revenue generated by the tariff reduction assumption. Tariff reductions in Latin America and the Caribbean, which have high rice import taxes, resulted in a positive consumer surplus and net welfare despite decreased tariff revenue. Third, the results show that, while India’s rice exports will decline substantially, other rice-producing and exporting countries or regions, such as East Asia-Pacific, Europe–Central Asia, South Asia, and the United States, will significantly increase their exports to importing countries, indicating a significant trade diversion. This will lead to rises in welfare when the rest of the world reduces tariffs, resulting in more exports. Despite increasing global consumer and producer surpluses, net welfare falls due to the considerable impact of tariff revenue losses.

## 6. Conclusions

This study revealed that India’s rice export ban, intended to provide price stabilization for Indian consumers, did not increase from local consumer surplus and significantly impacted India’s rice producers. While India experienced a loss in welfare, the rice-importing regions of the world, particularly Sub-Saharan Africa, Europe–Central Asia, and the GCC countries, suffered losses due to negative tariff revenues and rising consumer prices. As indicated in Table 13, there was a total global net welfare loss of USD 1.8 billion, of which USD 1 billion occurred in India. These findings indicate that the export ban policy hurts India more than the rest of the world, in contrast to its intent. The losses in non-Indian countries are 40% from reduced tariffs and 60% from lost consumer/producer surplus.

We studied several potential global reactions to the ban. To mitigate the negative net welfare in non-Indian countries from India’s export ban, a 75% decrease in rice import tariffs would be required. This mitigation of the negative impact is achieved through a significant increase in producer and consumer surplus while facing a significant drop in tariff revenues.

This study found that countries in Sub-Saharan Africa, the MENA, the GCC, and Europe–Central Asia are the most vulnerable to rice export volatility. Though India banned the export of non-Basmati rice to reduce the domestic market price, the consumer surplus is not increasing, and the local producer surplus is reduced. The global welfare impact is contingent on the magnitude of the response by importing countries. Strengthening rice trade diversification and maximizing rice source stability would significantly influence the countries or region’s overall welfare.

Our simulation technique provides an impartial evaluation of the impact of trade policies and regulatory circumstances. Nonetheless, there are limitations to our study, and specific improvements can be made for a more comprehensive assessment. Our study does not consider long-term modifications that may alter supply and pricing in the long run. Further research may include using a global dynamic computable general equilibrium modeling (CGE) to capture the economy-wide reactions to changes in exports, tariff policies, technology, or other external factors (notably climate change). Additional analysis should also focus on the distributional impacts on food trade and other food security implications, as well as on who is impacted (e.g., rural vs. urban, large vs. small producers).

## Figures and Tables

**Table 1:** Countries and regions and their abbreviations used in this study.

Countries	Abbreviations Used
Canada	Canada
China	China
India	India
United States	USA
Gulf Cooperation Council Counties (Saudi Arabia, UAE, Oman, Kuwait, Qatar, and Bahrain)	GCC
East Asia-Pacific	EAS-Pacific
Europe-Central Asia	EU-CAS
EU28-EU members (Inc. UK)	EU 28 (Inc. UK)
Latin America-Caribbean	LA-CN
Middle East–North Africa (Exc. GCC)	MENA (Exc. GCC)
South Asia-excluding India	SAS
Sub-Saharan Africa	SSA

Source: Authors based on World Integrated Trade Simulation (WITS) countries’ aggregation [34].

**Table 2:** Bilateral Rice Trade at World Price in 1000 US Dollars.

	*Destination*													
		Canada	China	EAS-Pacific	EU 28 (Inc. UK)	EU-CAS	GCC	India	LA-CN	MENA (Exc. GCC)	SAS	SSA	USA	Total Exports
***Origin***	Canada	0	0	103	208	222	21	0	105	113	0	335	13,344	14,452
	China	3054	0	344,466	20,640	100,440	4981	0	841	29,775	41,745	116,014	24,526	686,482
	East Asia-Pacific	83,137	1,415,738	2,758,737	609,103	669,241	111,032	1156	23,597	91,464	1386	1,040,961	633,549	7,439,101
	European Union 28 (Inc. UK)	4306	91	12,057	1,668,975	1,771,703	7316	681	10,831	49,056	102	11,470	27,476	3,564,065
	Europe–Central Asia	4594	121	20,449	1,687,877	1,876,732	8028	841	12,023	61,113	102	21,899	28,866	3,722,645
	Gulf Cooperation Council Countries	254	0	78	564	2218	42,501	209	4	40	594	103,918	6198	156,578
	India	71,687	372,556	775,026	374,087	537,563	1,594,474	0	15,200	110,067	534,466	2,359,103	226,064	6,970,293
	Latin America–Caribbean	3831	0	1353	265,057	307,802	5711	0	751,648	5700	0	80,682	43,619	1,465,404
	Middle East–North Africa (Exc. GCC)	1205	0	275	6131	5355	43,638	192	4	1693	0	5257	213	63,963
	South Asia	15,615	399,032	181,382	514,196	540,486	211,995	371	2878	8601	12,487	364,794	41,276	2,293,115
	Sub-Saharan Africa	42	0	40	135	140	262	55	107	29	0	246,038	95	246,942
	United States	193,526	11	523,361	47,695	58,299	115,848	303	623,038	77,950	2005	31,016	0	1,673,052
	Total imports	381,251	2,187,549	4,617,326	5,194,670	5,870,201	2,145,808	3809	1,440,276	435,601	592,887	4,381,487	1,045,226	28,296,091

Source: Authors based on World Integrated Trade Simulation (WITS) Trade Data [34].

**Table 3:** Bilateral Rice Import Tariffs rates for selected regions (%).

	*Destination*												
		Canada	China	EAS-Pacific	EU 28 (Inc. UK)	EU-CAS	GCC	India	LA-CN	MENA (Exc. GCC)	SAS	SSA	USA
***Origin***	Canada	1.00	1.00	1.01	1.00	1.12	1.00	1.00	1.86	1.89	1.00	1.95	1.11
	China	1.00	1.00	1.22	1.00	1.41	1.00	1.00	1.62	2.10	2.01	1.40	1.11
	EAS-Pacific	1.00	1.48	1.38	1.00	1.37	1.00	1.70	1.61	1.42	1.98	1.48	1.11
	EU 28 (Inc. UK)	1.00	1.65	1.02	1.00	1.35	1.00	1.74	1.60	1.54	1.69	1.70	1.11
	EU-CAS	1.00	1.65	1.08	1.00	1.30	1.00	1.74	1.66	1.47	1.69	1.65	1.11
	GCC	1.00	1.00	1.16	1.00	1.29	1.00	1.70	1.99	1.90	1.70	1.91	1.11
	India	1.00	1.11	1.34	1.00	1.29	1.00	1.00	1.87	1.43	1.59	1.44	1.11
	LA-CN	1.00	1.00	1.01	1.00	1.42	1.00	1.80	1.69	1.23	1.67	1.44	1.11
	MENA (Exc. GCC)	1.00	1.00	1.01	1.00	1.29	1.00	1.70	1.99	1.06	1.50	1.64	1.11
	SAS	1.00	1.47	1.38	1.00	1.23	1.00	1.79	1.83	1.41	1.59	1.50	1.11
	SSA	1.00	1.00	1.18	1.00	1.08	1.00	1.70	1.00	1.18	1.70	1.85	1.11
	USA	1.00	1.65	1.06	1.00	1.26	1.00	1.70	1.69	1.35	1.64	1.68	1.11

Source: Authors collected from the World Integrated Trade Simulation (WITS) tariff database [35].

**Table 4:** Import Demand, Export Supply, and Substitution Elasticities by Region.

Countries	Composite Demand	Export Supply Elasticity	Substitution Elasticity
Canada	−0.95	0.5	5
China	−1.05	0.5	5
East Asia-Pacific	−0.97	1.5	5
European Union 28 (Inc. UK)	−0.97	0.5	5
Europe–Central Asia	−0.94	0.5	5
Gulf Cooperation Council Countries	−0.95	0.5	5
India	−0.98	1.5	5
Latin America–Caribbean	−0.94	0.5	5
Middle East–North Africa (Exc. GCC)	−0.95	0.5	5
South Asia	−0.96	1.5	5
Sub-Saharan Africa	−0.95	0.5	5
United States	−1.04	1.5	5

Source: Grübler et al. [36] and Francois and Hall [28,29].

**Table 5:** Scenario 1. Welfare Effect and Percentage Price Changes. Base Scenario—India Export Ban of Rice (Voluntary Export Restriction).

	*Welfare in 1000 US Dollars*				*Price and Output (Percentage)*		
Countries	Producer Surplus	Consumer Surplus	Tariff Revenue	Net Welfare Effect	Change in Overall Consumer Prices	Change in Output	Producer Price for Home Good
	A	B	C	D = A + B + C	Percent	Percent	Percent
Canada	179	−6113	0	−5934	2%	1%	1%
China	9182	−48,136	−495	−39,450	2%	1%	1%
East Asia-Pacific	92,280	−99,787	−50,202	−57,709	2%	1%	1%
European Union 28 (Inc. UK)	31,724	−60,712	0	−28,988	1%	0%	1%
Europe–Central Asia	33,317	−94,573	−25,864	−87,120	1%	0%	1%
Gulf Cooperation Council Countries	2990	−65,091	0	−62,100	3%	1%	2%
India	−1,041,003	−77	0	−1,041,079	1%	−9%	−17%
Latin America–Caribbean	12,912	−24,642	−2068	−13,799	1%	0%	1%
Middle East–North Africa (Exc. GCC)	1207	−11,018	−8475	−18,286	2%	1%	2%
South Asia	28,579	−32,369	−61,447	−65,236	3%	1%	1%
Sub-Saharan Africa	4575	−165,781	−192,684	−353,890	3%	1%	2%
United States	18,348	−20,030	−4749	−6431	2%	1%	1%
Total	−805,710	−628,329	−345,984	−1,780,022			

**Table 6:** Scenario 1. Change in Percentages of the Rice Quantity Traded.

	*Destination*												
		Canada	China	EAS-Pacific	EU 28 (Inc. UK)	EU-CAS	GCC	India	LA-CN	MENA (Exc. GCC)	SAS	SSA	USA
***Origin***	Canada	0.0	0.0	0.4	−1.5	−1.3	5.9	0.0	−2.1	0.7	0.0	4.0	0.6
	China	−0.2	0.0	−0.1	−2.0	−1.8	5.4	0.0	−2.6	0.2	6.7	3.5	0.1
	East Asia-Pacific	0.3	−0.1	0.4	−1.5	−1.2	6.0	−1.5	−2.1	0.8	7.2	4.0	0.6
	European Union 28 (Inc. UK)	2.0	1.7	2.1	0.2	0.5	7.7	0.2	−0.3	2.5	9.0	5.7	2.3
	Europe–Central Asia	2.0	1.6	2.1	0.2	0.4	7.6	0.2	−0.4	2.4	8.9	5.7	2.3
	Gulf Cooperation Council Countries	−3.1	0.0	−3.0	−4.8	−4.6	2.6	−4.8	−5.4	−2.6	3.9	0.7	−2.7
	India	−11.4	−11.8	−11.4	−13.2	−13.0	−5.8	0.0	−13.8	−11.0	−4.5	−7.7	−11.1
	Latin America–Caribbean	2.0	0.0	2.1	0.3	0.5	7.7	0.3	−0.3	2.5	9.0	5.8	2.4
	Middle East–North Africa (Exc. GCC)	−2.9	0.0	−2.9	−4.7	−4.5	2.7	−4.7	−5.3	−2.5	0.0	0.8	−2.6
	South Asia	0.3	−0.1	0.4	−1.5	−1.3	5.9	−1.5	−2.1	0.7	7.2	4.0	0.6
	Sub-Saharan Africa	−2.8	0.0	−2.7	−4.5	−4.3	2.9	−4.6	−5.1	−2.3	0.0	0.9	−2.4
	United States	1.0	0.7	1.1	−0.8	−0.5	6.7	−0.8	−1.3	1.5	8.0	4.7	0.0

**Table 7:** Scenario 2. Welfare Effect and Percentage Price Changes (Importing Countries Response to India Rice Export Ban by 25% Reduction in Tariffs).

	*Welfare in 1000 US Dollars*				*Price and Output (Percentage)*		
Countries	Producer Surplus	Consumer Surplus	Tariff Revenue	Net Welfare Effect	Change in Overall Consumer Prices	Change in Output	Producer Price for Home Good
	A	B	C	D = A + B + C	Percent	Percent	Percent
Canada	657	−14,441	0	−13,784	4%	2%	4%
China	38,391	71,239	−153,941	−44,310	−2%	3%	6%
East Asia-Pacific	451,936	24,078	−283,121	192,893	0%	3%	6%
European Union 28 (Inc. UK)	142,478	−215,167	0	−72,689	4%	2%	4%
Europe–Central Asia	136,669	140,018	−378,939	−102,251	−2%	2%	4%
Gulf Cooperation Council Countries	13,744	−64,576	0	−50,832	3%	4%	9%
India	−1,090,588	423	−460	−1,090,624	−6%	−9%	−18%
Latin America–Caribbean	72,220	159,717	−171,455	60,483	−6%	2%	5%
Middle East–North Africa (Exc. GCC)	1875	16,363	−35,612	−17,374	−3%	1%	3%
South Asia	107,897	−12,217	−68,225	27,455	1%	2%	5%
Sub-Saharan Africa	29,046	14,702	−392,768	−349,019	0%	6%	11%
United States	54,774	−33,174	−25,960	−4360	3%	2%	3%
Total	−40,900	86,965	−1,510,480	−1,464,415			

**Table 8:** Scenario 2. Change in Percentages of the Rice Quantity Traded.

	*Destination*												
		Canada	China	EAS-Pacific	EU 28 (Inc. UK)	EU-CAS	GCC	India	LA-CN	MENA (Exc. GCC)	SAS	SSA	USA
***Origin***	Canada	0.0	0.0	−22.8	−6.1	−15.7	−10.5	0.0	12.1	28.8	0.0	40.3	1.6
	China	−12.5	0.0	−5.4	−11.2	3.5	−15.6	0.0	−3.0	31.3	43.8	9.2	−3.4
	East Asia-Pacific	−14.0	4.8	5.7	−12.7	−0.6	−17.1	0.2	−4.9	−0.2	41.5	12.9	−4.8
	European Union 28 (Inc. UK)	−4.7	22.4	−18.8	−3.4	6.6	−7.8	10.3	3.1	15.6	38.4	32.8	4.2
	Europe–Central Asia	−3.1	23.9	−10.2	−1.8	4.4	−6.2	11.8	7.4	13.0	39.8	31.9	5.8
	Gulf Cooperation Council Countries	−27.9	0.0	−25.8	−26.6	−19.7	−30.9	−12.2	−1.3	11.1	18.0	20.8	−18.4
	India	3.2	−20.8	−13.4	4.5	−19.1	0.2	0.0	−37.7	−22.1	−6.7	−12.8	−0.7
	Latin America–Caribbean	−9.3	0.0	−24.6	−8.0	7.2	−12.3	8.8	3.3	−10.0	33.4	14.8	−0.2
	Middle East–North Africa (Exc. GCC)	0.5	0.0	−14.9	1.8	7.1	−2.5	13.3	23.6	−17.5	0.0	34.7	9.4
	South Asia	−7.7	10.0	11.7	−6.4	−5.6	−10.7	9.8	10.7	5.0	30.8	19.9	1.4
	Sub-Saharan Africa	−42.1	0.0	−37.5	−40.8	−54.1	−45.2	−25.0	−83.0	−46.1	0.0	5.9	−32.2
	United States	−0.9	25.8	−10.3	0.4	3.2	−4.0	12.0	10.8	7.3	39.5	35.3	0.0

**Table 9:** Scenario 3. Welfare Effect and Percentage Price Changes (Importing Countries Respond by Reducing Rice Imports Tariff from Non-Indian Countries by 50%).

	*Welfare in 1000 US Dollars*				*Price and Output (Percentage)*		
Countries	Producer Surplus	Consumer Surplus	Tariff Revenue	Net Welfare Effect	Change in Overall Consumer Prices	Change in Output	Producer Price for Home Good
	A	B	C	D = A + B + C	Percent	Percent	Percent
Canada	1179	−23,341	0	−22,163	6%	4%	8%
China	70,774	205,678	−347,169	−70,717	−6%	5%	10%
East Asia-Pacific	873,266	160,950	−565,492	468,724	−3%	5%	11%
European Union 28 (Inc. UK)	262,056	−384,803	0	−122,747	7%	4%	7%
Europe–Central Asia	247,267	396,238	−789,695	−146,190	−5%	3%	7%
Gulf Cooperation Council Countries	26,562	−63,710	0	−37,147	3%	8%	16%
India	−1,143,626	996	−1057	−1,143,687	−14%	−10%	−19%
Latin America–Caribbean	138,728	369,004	−388,030	119,701	−14%	5%	9%
Middle East–North Africa (Exc. GCC)	2544	46,755	−73,483	−24,183	−7%	2%	4%
South Asia	196,165	9354	−84,621	120,898	−1%	4%	8%
Sub-Saharan Africa	60,924	212,978	−674,351	−400,450	−3%	12%	23%
United States	92,518	−47,796	−47,180	−2459	4%	3%	5%
Total	828,358	882,302	−2,971,079	−1,260,419			

**Table 10:** Scenario 3. Percentages Change of the Rice Quantity Traded.

	*Destination*												
		Canada	China	EAS-Pacific	EU 28 (Inc. UK)	EU-CAS	GCC	India	LA-CN	MENA (Exc. GCC)	SAS	SSA	USA
***Origin***	Canada	0.0	0.0	−47.8	−11.1	−31.3	−28.1	0.0	27.2	58.6	0.0	78.6	2.8
	China	−26.2	0.0	−11.2	−21.4	9.5	−38.4	0.0	−2.7	65.3	84.1	15.3	−7.0
	East Asia-Pacific	−30.2	10.2	11.5	−25.4	0.3	−42.4	2.9	−7.0	−0.9	79.0	22.6	−10.8
	European Union 28 (Inc. UK)	−12.0	44.1	−41.3	−7.3	13.2	−24.3	20.9	6.7	29.3	69.4	61.3	6.4
	Europe–Central Asia	−8.6	46.8	−23.4	−3.8	8.6	−20.8	23.6	15.5	23.9	72.2	59.2	9.7
	Gulf Cooperation Council Countries	−57.4	0.0	−51.9	−52.6	−36.4	−69.6	−18.7	5.4	27.7	34.3	44.0	−36.7
	India	18.9	−30.6	−15.7	23.6	−25.5	6.6	0.0	−62.9	−33.8	−9.2	−18.2	10.7
	Latin America–Caribbean	−22.2	0.0	−54.0	−17.4	14.3	−34.4	18.2	7.6	−23.7	59.4	24.2	−3.2
	Middle East–North Africa (Exc. GCC)	4.4	0.0	−27.6	9.2	18.5	−7.8	30.3	51.9	−33.5	0.0	68.7	22.0
	South Asia	−16.2	20.6	23.6	−11.5	−10.0	−28.5	21.9	24.5	9.7	56.0	36.7	2.4
	Sub-Saharan Africa	−92.5	0.0	−80.0	−87.7	−114.0	−104.7	−46.6	−174.3	−98.1	0.0	12.1	−70.0
	United States	−2.5	51.7	−22.2	2.3	7.2	−14.7	24.9	23.2	13.1	72.2	67.0	0.0

**Table 11:** Scenario 4. Welfare Effect and Percentage Price Changes (Importing Countries Respond by Reducing Rice Import Tariffs from Non-Indian Countries by 75%).

	*Welfare in 1000 US Dollars*				*Price and Output (Percentage)*		
Countries	Producer Surplus	Consumer Surplus	Tariff Revenue	Net Welfare Effect	Change in Overall Consumer Prices	Change in Output	Producer Price for Home Good
	A	B	C	D = A + B + C	Percent	Percent	Percent
Canada	1751	−32,840	0	−31,089	8%	6%	12%
China	106,821	359,011	−589,367	−123,535	−11%	7%	15%
East Asia-Pacific	1,369,817	315,184	−908,620	776,381	−5%	8%	16%
European Union 28 (Inc. UK)	391,471	−571,733	0	−180,262	10%	5%	11%
Europe–Central Asia	365,818	678,446	−1,267,589	−223,325	−8%	5%	10%
Gulf Cooperation Council Countries	42,085	−62,373	0	−20,287	3%	13%	25%
India	−1,200,561	1654	−1814	−1,200,721	−23%	−10%	−20%
Latin America–Caribbean	213,950	607,339	−659,558	161,732	−23%	7%	14%
Middle East–North Africa (Exc. GCC)	3212	80,860	−124,361	−40,289	−12%	2%	5%
South Asia	294,438	33,304	−113,126	214,616	−3%	6%	12%
Sub-Saharan Africa	104,125	437,464	−1,061,868	−520,280	−6%	19%	38%
United States	131,142	−64,140	−68,090	−1088	5%	4%	7%
Total	1,824,070	1,782,175	−4,794,393	−1,188,148			

**Table 12:** Scenario 4. Percentages Change of the Rice Quantity Traded.

	*Destination*												
		Canada	China	EAS-Pacific	EU 28 (Inc. UK)	EU-CAS	GCC	India	LA-CN	MENA (Exc. GCC)	SAS	SSA	USA
***Origin***	Canada	0.0	0.0	−75.0	−16.6	−48.0	−47.2	0.0	43.4	90.2	0.0	119.0	4.0
	China	−41.4	0.0	−17.5	−32.8	16.3	−63.4	0.0	−1.5	102.6	127.9	21.9	−11.0
	East Asia-Pacific	−48.4	16.2	17.9	−39.8	1.8	−70.4	6.7	−8.2	−1.2	120.3	33.3	−17.4
	European Union 28 (Inc. UK)	−19.9	66.7	−65.7	−11.3	20.0	−41.9	32.0	10.6	43.8	102.2	91.1	8.9
	Europe–Central Asia	−14.4	70.6	−37.8	−5.8	12.8	−36.4	35.7	23.8	35.1	106.0	87.6	14.0
	Gulf Cooperation Council Countries	−92.8	0.0	−81.9	−84.2	−54.9	−114.8	−24.0	15.7	47.8	53.3	71.1	−58.5
	India	35.6	−41.3	−18.3	44.2	−32.1	13.6	0.0	−89.6	−46.3	−11.8	−24.3	23.3
	Latin America–Caribbean	−37.0	0.0	−86.6	−28.3	21.9	−58.9	28.6	12.6	−38.8	87.4	33.9	−6.8
	Middle East–North Africa (Exc. GCC)	8.8	0.0	−41.2	17.4	29.6	−13.2	46.2	79.4	−50.8	0.0	102.5	35.5
	South Asia	−25.4	31.7	36.2	−16.8	−14.7	−47.4	35.0	39.6	14.6	82.8	54.5	3.9
	Sub-Saharan Africa	−158.8	0.0	−133.4	−150.1	−188.0	−180.7	−69.6	−283.9	−161.4	0.0	19.9	−119.6
	United States	−3.6	78.2	−34.6	5.1	11.4	−25.5	37.7	35.8	19.0	106.3	99.6	0.0

**Table 13:** Global Welfare Impact of India’s Ban on Non-Basmati Rice Exports.

Welfare Effect	Scenario 1	Scenario 2	Scenario 3	Scenario 4
Producer Surplus (PS)	−805,710	−40,900	828,358	1,824,070
Consumer Surplus (CS)	−628,329	86,965	882,302	1,782,175
Economic Surplus (PS + CS)	−1,434,039	46,066	1,710,660	3,606,245
Tariff Revenue Change	−345,984	−1,510,480	−2,971,079	−4,794,393
Net welfare	−1,780,022	−1,464,415	−1,260,419	−1,188,148

## Data Availability

The original data used in the study are openly available in the World Integrated Trade Solution (WITS) website: https://wits.worldbank.org/.
